# Sex Based Comparison of Health Self-Perception, Sleep, Anxiety, and Body Composition Among University Students

**DOI:** 10.3389/ijph.2025.1608551

**Published:** 2025-09-26

**Authors:** Sondos Basheer, Hadia Radwan, Veena Raigangar, Falak Zeb, Ayat Zamrik, Raghda Adi, Tala Faraj, Woroud Chaker, Tareq Osaili, Hayder Hasan

**Affiliations:** ^1^ Department of Clinical Nutrition and Dietetics, College of Health Sciences, University of Sharjah, Sharjah, United Arab Emirates; ^2^ Nutrition and Food Research Group, Research Institute for Medical and Health Sciences, University of Sharjah, Sharjah, United Arab Emirates; ^3^ School of Sport and Health Sciences, University of Brighton, Brighton, United Kingdom; ^4^ Department of Nutrition and Food Technology, Faculty of Agriculture, University of Science and Technology, Irbid, Jordan

**Keywords:** health self-perception, self-rated health, body composition indicators, sleep quality, anxiety

## Abstract

**Objectives:**

Health self-perception (HSP) refers to an individual’s subjective evaluation of their overall health, encompassing both physical and psychological dimensions. A number of factors, such as body composition, anxiety levels, and sleep quality, can have an impact on HSP among university students. There is a limited research investigating the combined association between HSP, sleep quality, and anxiety status in this demographic, despite the fact that each of these characteristics has been examined separately in relation to students’ health. Furthermore, students’ perceptions and reporting of their health may be influenced by contextual and cultural factors, particularly in the United Arab Emirates (UAE). This study aimed to explore the association of HSP with anthropometric measurements, sleep quality, and anxiety status and to compare these associations between female and male university students.

**Methods:**

This cross-sectional study recruited 390 university students (198 males and 192 females), aged 18–25 years. A validated questionnaire was used for determining the sleep quality, anxiety and HSP while body composition was measured by using body analyzer.

**Results:**

Majority (59%) of the students reported positive HSP which was significantly associated with higher sleep quality and lower state anxiety scores compared to negative HSP group. Females were more likely to have negative HSP compared to males (p < 0.001). Moreover, the State-Trait Anxiety Inventory (STAI) score, PBF, FM, and VFR were significantly lower while sleep quality score and FFM were significantly higher among positive HSP group.

**Conclusion:**

This study highlighted that good sleep quality, low anxiety levels, and healthy body composition are correlated with positive HSP and were significantly dependent on sex. A comprehensive health program is essential to improve sleep quality and anxiety status to promote good HSP among university students.

## Introduction

Health self-perception (HSP) is an individual’s assessment that allows a person to assess their overall state of health which includes both physical and psychological dimensions [1]. The major drivers that can affect the individual’s health status are the economic and social context (40%), health behavior (40%), clinical care (20%) and physical environment (10%) [2], as well as the genetics that has an important impact on disease development, life quality and mortality [3]. In addition, each of these has the potential to affect not just the general health but also other drivers [2]. Numerous studies have identified several factors affecting self-reported health (SRH) including socioeconomic characteristics, employment position, and educational attainment [4]. In addition, sex, education, culture, personality, and even generation could explain the variation in HSP [5].

A previous cross-sectional study has found that physical health including BMI, skeletal muscle function, mobility, number of chronic diseases, and use of drugs and alcohol has a significant effect on one’s health status perception, both mental and cognitive functioning, where depression and impaired cognitive capabilities were associated with lower HSP rating [6]. Accordingly, a recent study has demonstrated that HSP was lower among those with higher ages, body mass indices, and chronic conditions (cancer, diabetes, ischemic heart disease, depression, and anxiety), while better HSP was linked to moderate alcohol use and a high degree of physical exercise [5]. In regard to gender differences in health perceptions, it was reported in a school-based study where data was collected from the national health surveys, that girls tend to have a greater negative health perception rate compared to boys (33% vs. 19%, P < 0.001) [7]. Likewise, a longitudinal study of a random sample of 1,516 adults has found that 35.6% of the participants perceived their health as negative, where 60% of the females indicated a negative health perception compared with 40% of males [5].

Sleep quality is one of the key components of a sleeping pattern that is considered an important factor that affect someone’s health perception due to its numerous effects on the quality of life such as physical and mental function, and psychological impacts [8]. A recent study indicated that poor sleep quality has the strongest association with poor HSP [9]. Similarly, a study that has targeted healthcare students in China found that there was a significant direct association between sleep quality, depression, and HSP [10]. Moreover, a previous study targeting healthcare students in Saudi Arabia, indicated that the likelihood of poor sleep quality was significantly correlated with subjective stress and anxiety [11]. Another key component of sleep wellness is sleeping duration. In comparison to normal or mid-range sleep, both short and lengthy sleep durations have been associated with worse health, as measured by various risk markers, clinical evaluations, or self-rated (subjective) health (SRH). Therefore, a cross-sectional study suggests that people who slept for a long time with poor quality had very low SRH, whereas people who slept for a long time might be protected from having low SRH by having good sleep quality [12]. To add up, the number of studies targeting both dimensions of sleep health; sleep quality and sleep quantity still limited especially the region-specific studies, particularly in the Middle East, where cultural, social, and occupational contexts may uniquely influence health and wellbeing. In UAE, a new study targeting healthcare professionals was recently conducted to address this gap by examining sleep health, wellbeing, and optimism in relation to SRH. It was demonstrated that sleep circadian regularity (p = 0.009) Wellbeing (p < 0.001), and optimism (p = 0.004) were significant predictors of SRH, accounting for 10% of the variance in SRH (R2 = 0.103), which reflects the correlation between those variables and SRH [13].

Moreover, anxiety may also be an important factor that affects health status. Anxiety is a future-focused mood state that includes a sophisticated cognitive, affective, physiological, and behavioral response system related to being ready for the events or situations that are expected to happen or that are viewed as dangerous [14]. The number of people suffering from anxiety disorders is increasing worldwide. An estimated 301 million people worldwide, or 4.05% of the total population, suffer from an anxiety illness [15]. In the United Arab Emirates (UAE), 28% of the adolescent population had anxiety [16], while 32.2% of University of Sharjah students had moderate to severe anxiety post-lock-down of COVID-19 which was significantly correlated with poor sleep quality [17]. Mahmoud et al. [18], suggested that anxiety is reported in 56.4% adult population with a higher prevalence in women compared to men, which in terms shows that the prevalence of anxiety is higher than other disorders in the UAE.

Two types of psychological anxiety are identified in the literature: state and trait anxiety. State anxiety is a fleeting sensation of anxiety, which can be brought on by natural causes or by experimental methods (such as anxiogenic tasks or manipulations) [19], while trait anxiety is the propensity to perceive circumstances as threatening, steer clear of anxiety-inducing situations, and exhibit elevated baseline physiological arousal [20]. Moreover, anxiety can lead to more serious psychiatric problems like major depression, suicidal thoughts, and substance use disorder which affects an individual’s perceived health [21]. These facts were confirmed by Hossain et al. [22], where it was reported that poor HSP was significantly associated with depression and anxiety among university students in Bangladesh which increased physiological dysfunction in later life. There is also evidence that both depression symptoms and poor SRH could lead to each other, however, the effect of poor SRH was stronger [23]. Not only anxiety and sleep health but also nutritional status has been considered a contributing factor that affects self-rated health whereas normal body composition indicators may indicate good HSP [24]. Numerous studies have investigated the relationship between obesity and SRH, with varying results. A study conducted on older adults in Korea, revealed that there was a negative association between BMI and SRH in males, while in women positive association was seen. Regardless of BMI, those who have perceived their weight as higher or lower than their real weight had a higher risk of poor SRH [25]. Another study on Korean adults has found that there is a significant J-shaped association between BMI and poor SRH, which is highly dependent on age, as the association between underweight and poor SRH was stronger in younger adults and in contrast higher association was found between overweight and poor SRH in older ages [26]. However, a European data analysis of 299,846 participants concluded that poorer SRH was associated with higher BMI assuming that participants perceived the effect of their weight on health correctly [27].

In fact, country-specific value systems are especially necessary in nations with a majority of Muslims, like the United Arab Emirates (UAE). As UAE has unique health needs and cultural influences on health perception and health status. Research on health-related quality of life revealed that Emiratis valued health status differently from other cultural groups. Also, cultural factors play a significant role in shaping health perceptions and attitudes towards mental health issues [28, 29]. We hypothesize that sleep quality, anxiety, and body composition indicators influence HSP which has not been explored among university students in the UAE. Therefore, this study aims to explore the association of HSP with anthropometric parameters, sleep quality, and anxiety status among university students.

## Methods

### Study Design and Settings

In this cross-sectional study, a convenient sample of 390 students (both health-related and non-health-related major) was recruited from the University of Sharjah (UOS), Sharjah, UAE from February until March 2020, as a part of voluntary research project with an emphasis on contributing to academic research and gaining practical insights into research process as well they represent a critical population for studying HSP, sleep quality and anxiety–which are factors that are highly relevant during this life stage. The inclusion criteria were male and female university students from different nationalities aged 18–25 years who were free from any chronic diseases.

### Ethical Approval

The study was approved by the Research Ethics Committee of the University of Sharjah (REC-20-03-07-03-S), and written consent was taken from all the participants before enrolling in the study.

### Data Collection

The validated multicomponent questionnaire consisted of socio-demographic questions (such as age, sex, marital status, nationality, study major, educational level, and living place), diet quality and weight satisfaction, a sleep quality questionnaire [30], and an anxiety scale [31], were used for data collection. Health self-perception (HSP) was measured using the first question of the survey “How do you perceive your health?” which has the following possible answers: excellent, good, fair, and poor. To compare with other studies, we recorded this variable as positive (excellent, and good) HSP or negative (fair and poor) HSP.

### Sleep Quality and Anxiety Status

Sleep-quality questionnaire (SQQ) is a self-reported questionnaire with 9 items with a 4-point Likert scale response for each item to generate the sleep score refers to the total score of the SQQ [30]. The sum of the responses generated a score, which was interpreted as below:

**Table:** 

Score	Interpretation
0–9	Sleep problems seem to be severe Should daintily try to get some help
10–18	There are some sleep problems It’s important to examine sleep habits and see how to make changes
19–27	Sleep is in good shape, but there are still many steps that can be taken to make it even better
28–36	Sleep is in great shape

A self-administered State-trait anxiety inventory (STAI) questionnaire was used to measure the anxiety status. In which the State anxiety (SA) and trait anxiety (TA) are measured by the STAI, which has 40 Likert-type items, 20 of which are for the SA scale and 20 of which are for the TA scale [31].

Questions 1–20 assess state (STAI state) anxiety which indicates the intensity of anxiety feeling “right now”, and questions 21–40 assess trait (STAI trait) anxiety which reports the frequency of anxiety feelings “in general”. Cutoffs for levels of anxiety were set for both state and trait anxiety with scores of 20–37 for no or low, 38–44 for moderate, and 45–80 for high anxiety.

The validity and reliability of the English version of the STAI questionnaire were tested through confirmatory factor analysis in which a total of 294 participants filled out the State-Trait Anxiety Inventory, the Cognitive Test Anxiety, and the Positive and Negative Affect Schedule. Results showed that validity of both latent versions of the STAI, two-factor (with State anxiety and State Calmness factors) and a bi-factor solution (with general state anxiety, positive wording, and negative wording factors), additionally, reliability analysis of state anxiety showed a consistency within the variables [32, 33]. The validity and reliability of the Arabic version of the STAI questionnaire were evaluated in a cross-sectional study in Saudi Arabia, in which the analysis showed that the Arabic version of the STAI-Y was unidimensional, and its internal consistency reliability for the state and trait subscales was high [32].

### Body Composition Indicators

The student’s body weight (kg) and height (cm) were measured by using a Seca 220 Telescopic with a measuring rod while the waist circumference (WC) in (cm) was measured by the Seca measuring tape [34]. Percent body fat (PBF), muscle mass (MM), bone mass (BM), fat-free mass (FFM), and visceral fat rating (VFR) were measured by the TANITA SC-331S body composition analyzer. The actual body mass index (BMI) was calculated as weight (kg)/[height (m)]^2^ based on the measured weight and height of the participants, while the perceived BMI (kg/m^2^) was calculated from the reported weight and height of the participants. The participants were classified as underweight (BMI <18.5 kg/m^2^), normal weight (BMI 18.5–24.99 kg/m^2^), and overweight/obese (BMI ≥25 kg/m^2^).

### Data Analysis

Data analysis was conducted by using Statistical Package for the Social Sciences version 21.0 (SPSS, Chicago, IL, USA). The normality of the distribution of the data was checked by the Kolmogorov-Smirnov test. Frequencies, percentages, medians, and interquartile ranges were calculated, and associations were determined using the Chi-square. The Mann-Whitney U test was used to compare the studied numerical parameters. Spearman’s correlation and binary logistic regression analysis were applied to explore the relationships of the HSP with sleep score and anxiety status. The level of significance was set at p < 0.05.

## Results

### Socio-Demographic Characteristics of the Participants

A total of 390 students (males/females: 198/192) from the UOS participated in this study. About 66% of them were below the age of 20 years (range 18–25) and the majority were single and Arabs. More than half of the participants (55%) were studying in health-related majors and about 36% were first-year students while 72.6% were living with their families. About 59% of them perceived their health as positive whereas 41% perceived their health as negative. About 70% of the students did not follow any strict diet, and approximately 55% were not satisfied with their body weight (Table 1).

**TABLE 1:** Socio-demographic characteristics of the participants (n = 390) (Sharjah, United Arab Emirates, 2020).

Variables	All (n = 390) %(N)	Females (n = 192) %(N)	Males (n = 198) %(N)
Age group			
≤20 years	66.2 (258)	67.7 (130)	64.6 (128)
≥21 years	33.8 (132)	32.3 (62)	35.4 (70)
Marital status			
Single	97.4 (380)	97.4 (187)	97.5 (193)
Married	2.6 (10)	2.6 (5)	2.5 (5)
Nationality			
Arab	89.5 (349)	87.5 (168)	91.4 (181)
Non-Arab	10.5 (41)	12.5 (24)	8.6 (17)
Study major			
Health-related	54.9 (214)	75.5 (145)	65.2 (129)
Non-health related	45.1 (176)	24.5 (47)	34.8 (69)
Education Level			
First-year	35.9 (140)	34.4 (66)	37.4 (74)
Second year	24.9 (97)	26.0 (50)	23.7 (47)
Third year	13.8 (54)	15.1 (29)	12.6 (25)
Fourth year	25.43 (99)	24.4 (47)	26.3 (52)
Place of residence			
In the university dorms	24.1 (94)	21.9 (42)	26.3 (52)
In a rented apartment	3.3 (13)	3.1 (6)	3.5 (7)
With the family	72.6 (283)	75 (144)	70.2 (139)
Do you follow a diet and why?			
To lose weight	23.6 (92)	24.0 (46)	23.2 (46)
To gain weight	6.9 (27)	2.6 (5)	11.1 (22)
I do not follow any diet	69.5 (271)	73.4 (141)	65.7 (130)
How do you perceive your health (HSP)?			
Positive	59 (230)	51.0 (98)	66.6 (132)
Negative	41 (160)	49.0 (94)	33.4 (66)
Are you satisfied with your weight?			
No	54.6 (213)	56.8 (109)	52.5 (104)
Yes	45.4 (177)	43.2 (83)	47.5 (94)

### Comparison of Sleep Score, Anxiety Scores, and Anthropometric Measurements Between Male and Female

Sleep and anxiety scores, along with anthropometric measurements, were compared according to sex (Table 2). Males had significantly higher WC, FFM, MM, VFR, actual and perceived BMI, and STAI trait scores, while females had significantly higher PBF, fat mass, and STAI state scores. Males and females did not demonstrate differences in sleep scores. On the other hand, males showed higher STAI trait scores [48 (9.25)] than STAI state scores [44 (13)], whereas females had equal STAI trait and STAI state scores [46 (9) and 46 (14), respectively].

**TABLE 2:** Comparison of sleep scores, anxiety scores, and anthropometric measurements according to the sex (Sharjah, United Arab Emirates, 2020).

Variables	Total (n = 390)	Female (n = 192)	Male (n = 198)	p-value
WC (cm)	78.75 (18.00)	72.00 (13.00)	86.00 (18.25)	<0.001
PBF (%)	22.05 (13.75)	25.50 (11.05)	17.45 (11.60)	<0.001
Fat Mass (kg)	14.10 (11.55)	14.60 (9.98)	13.15 (13.03)	<0.001
FFM (kg)	50.45 (20.85)	42.40 (5.15)	63.05 (12.65)	<0.001
Muscle Mass (kg)	47.85 (19.83)	40.25 (4.95)	59.95 (12.05)	<0.001
VFR (%)	2.00 (3.00)	1.00 (1.00)	3.00 (5.00)	<0.001
Actual BMI (kg/m^2^)	23.60 (6.62)	22.20 (5.50)	25.10 (7.15)	<0.001
Perceived BMI (kg/m^2^)	23.16 (6.07)	21.95 (4.95)	24.61 (6.58)	<0.001
Sleep score	23.00 (8.00)	23.00 (8.75)	24.00 (8.00)	0.16
STAI State score	45.00 (15.00)	46.00 (14.00)	44.00 (13.00)	0.036
STAI Trait score	47.00 (10.00)	46.00 (9.00)	48.00 (9.25)	0.005

WC, waist circumference; PBF, percent body fat; FFM, fat free mass; VFP, visceral fat rating; BMI, body mass index; STAI, State-trait anxiety inventory.

### Association of Health Self-Perception With Socio-Demographic Characteristics

The association of HSP with sociodemographic variables and BMI is presented in Supplementary Table S1. The perception of health as positive or negative was independent of age, marital status, health-related majors, and educational level, whereas it was dependent on sex and BMI. Males were more likely to perceive their health as positive compared to females [57.4% vs. 42.6%, OR = 0.52; 95%CI: 0.34–0.78, p = 0.002]. Among those who perceived their health as positive, 60% had normal BMI and 40% had abnormal BMI. On the other hand, among those who negatively perceived their health, 45% had normal BMI and 55% had abnormal BMI [OR = 0.54; 95%CI: 0.36–0.82, p = 0.003].

### Comparison of Sleep Score, Anxiety Score, and Anthropometric Measurements Based on Sex and Categories of Health-self-perception

Associations between HSP and sleep score, anxiety score, and anthropometric measurements are presented (see Table 3). It shows that all participants who reported their health as positive had significantly higher sleep scores and FFM than those with negative HSP. Moreover, those who had positive HSP illustrated significantly lower STAI state scores, PBF, FM, and VFR compared to those with negative HSP. However, both actual and perceived BMI were non-significantly different in positive and negative groups. It was also noted that the WC, PBF, FM, VFR, actual and perceived BMI of male participants were significantly different between positive and negative HSF groups. Among female participants, sleep score, STAI state score, and STAI trait score were significantly different between positive and negative HSP groups.

**TABLE 3:** Comparison of sleep score, anxiety score, and anthropometric measurements according to the health-self-perception (Sharjah, United Arab Emirates, 2020).

Variables	All			Males			Females		
	Health self perception		P-value	Health self perception		P-value	Health self perception		P-value
	Positive median (IQR) N = 230	Negative median (IQR) N = 160		Positive median (IQR) N = 132	Negative median (IQR) N = 66		Positive median (IQR) N = 98	Negative median (IQR) N = 94	
Sleep score	24 (8)	22 (8.7)	0.002	24.0 (8.0)	22.5 (8.25)	0.30	24.0 (8.0)	22.0 (9.0)	0.002
STAI State Score	44 (13)	46 (15)	0.002	44.0 (14.0)	43.5 (14.0)	0.18	44.5 (13.0)	48.0 (14.0)	0.001
STAI Trait Score	46.5 (8)	47 (11)	0.46	48.0 (9.0)	47.0 (15.0)	0.59	45.0 (9.0)	47.0 (10.0)	0.019
WC (cm)	79.0 (15)	77.5 (23.5)	0.87	85.5 (14.0)	94.5 (24.7)	0.001	71.5 (12.2)	72.0 (13.0)	0.89
PBF (%)	19.3 (12.75)	24.8 (13.9)	<0.001	15.95 (9.4)	22.15 (15.8)	<0.001	25.8 (9.6)	25.45 (11.9)	0.43
Fat Mass (kg)	13.05 (10.5)	16.05 (14.0)	0.001	12.2 (10.3)	17.8 (19.1)	<0.001	14.65 (9.5)	14.6 (10.5)	0.56
FFM (kg)	53.45 (20.6)	47.5 (19.3)	0.011	62.05 (12.7)	64.25 (12.5)	0.59	42.4 (5.23)	42.45 (5.3)	0.88
Muscle Mass (kg)	50.75 (19.6)	45.1 (18.4)	0.11	58.95 (12.1)	61.05 (11.9)	0.59	40.2 (5.02)	40.35 (5.0)	0.89
VFR (%)	2.81 (3.1)	4.13 (4.9)	0.001	2.0 (4.0)	5.0 (8.2)	<0.001	1.0 (1.0)	1.0 (2.0)	0.36
Actual BMI (kg/m^2^)	23.5 (5.5)	24.1 (8.05)	0.13	24.3 (6.1)	27.35 (9.0)	<0.001	22.2 (5.05)	22.1 (5.77)	0.89
Perceived BMI (kg/m^2^)	22.87 (5)	23.48 (8.0)	0.13	23.52 (5.3)	26.83 (7.9)	<0.001	22.1 (4.7)	21.9 (5.7)	0.95

STAI, State-trait anxiety inventory; WC, waist circumference; PBF, percent body fat; FFM, fat free mass; VFR, visceral fat rating; BMI, body mass index.

### Correlation Analysis Between Sleep, STAI State, and STAI Trait Scores With Different Anthropometric Indices

Correlations of sleep, STAI State, and STAI Trait scores with various anthropometric indices are reported (see Table 4). In the negative HSP group, sleep score showed a highly significant negative correlation with the STAI State score (r = −0.41, p < 0.001). Whereas, in the positive HSP group, sleep score showed a significant negative correlation with the STAI State score (r = −0.37, p < 0.001), Fat mass (r = −0.13, p < 0.05), and VFR (r = −0.15, p < 0.05). The same group showed a significant positive correlation between STAI State score and WC (r = 0.15, p < 0.05). On the other hand, the positive HSP group, STAI Trait Score showed a significant negative correlation with the WC (r = −0.13, p < 0.05) and significant positive correlations with FFM (r = 0.13, p < 0.05) and MM (r = 0.13, p < 0.05). Furthermore, we have compared these correlations in females and male participants (Tables 5, 6).

**TABLE 4:** Correlations of sleep, State-trait Anxiety Inventory State, and State-trait Anxiety Inventory Trait Scores with anthropometric measures (Sharjah, United Arab Emirates, 2020).

Health perception categories		Sleep score	STAI state score	STAI trait score
Negative Health Perception group (n = 160)	BMI	0.07	—	—
	STAI State Score	−0.41**	—	—
	STAI Trait Score	−0.02	−0.01	—
	Waist circumference	0.13	−0.02	0.02
	Waist circumference	0.01	0.12	0.01
	Fat mass	0.06	0.08	0.03
	FFM	0.11	−0.02	−0.03
	Muscle mass	0.11	−0.02	−0.03
	Visceral fat rating	0.08	0.03	0.05
Positive Health Perception group (n = 230)	BMI	−0.11	—	—
	STAI State Score	−0.37**	—	—
	STAI Trait Score	−0.07	0.02	—
	Waist circumference	−0.10	0.06	0.08
	Waist circumference	−0.10	0.15*	−0.13*
	Fat mass	−0.13*	0.12	−0.03
	FFM	−0.001	−0.03	0.13*
	Muscle mass	−0.001	−0.03	0.13*
	Visceral fat rating	−0.15*	0.06	0.10

*p < 0.05, **p < 0.001.

**TABLE 5:** Correlations of sleep, State-trait Anxiety Inventory State, and State-trait Anxiety Inventory Trait Scores with anthropometric measures in females (n = 192) (Sharjah, United Arab Emirates, 2020).

Health perception categories		Sleep score	STAI state score	STAI trait score
Negative Health Perception group (n = 94)	BMI	−0.03	—	—
	STAI State Score	−0.34**	—	—
	STAI Trait Score	−0.02	−0.04	—
	Waist circumference	0.08	0.08	−0.07
	Percent body fat	0.02	0.14	−0.06
	Fat mass	0.01	0.15	−0.08
	FFM	−0.04	0.21*	−0.23*
	Muscle mass	−0.04	0.21*	−0.23*
	Visceral fat rating	0.03	0.15	−0.03
Positive Health Perception group (n = 98)	BMI	−0.05	—	—
	STAI State Score	−0.44**	—	—
	STAI Trait Score	−0.08	0.01	—
	Waist circumference	0.02	0.05	−0.05
	Percent body fat	−0.03	0.12	−0.06
	Fat mass	−0.04	0.09	−0.06
	FFM	−0.07	0.06	−0.01
	Muscle mass	−0.07	0.06	−0.01
	Visceral fat rating	−0.01	−0.01	0.04

*p < 0.05, **p < 0.001.

**TABLE 6:** Correlations of sleep, State-trait Anxiety Inventory State, and State-trait Anxiety Inventory Trait Scores with anthropometric measures in males (n = 198) (Sharjah, United Arab Emirates, 2020).

Health perception categories		Sleep score	STAI state score	STAI trait score
Negative Health Perception group (n = 66)	BMI	0.06	—	—
	STAI State Score	−0.50**	—	—
	STAI Trait Score	−0.05	0.01	—
	Waist circumference	0.03	0.06	0.18
	Percent body fat	0.03	0.03	0.12
	Fat mass	0.05	0.03	0.15
	FFM	0.08	0.07	0.16
	Muscle mass	0.08	0.07	0.17
	Visceral fat rating	0.02	0.05	0.15
Positive Health Perception group (n = 132)	BMI	−0.01	—	—
	STAI State Score	−0.31**	—	—
	STAI Trait Score	−0.11	0.03	—
	Waist circumference	−0.09	0.10	−0.03
	Percent body fat	−0.06	0.10	−0.02
	Fat mass	−0.04	0.11	−0.04
	FFM	0.05	0.08	−0.10
	Muscle mass	0.05	0.08	−0.10
	Visceral fat rating	−0.06	0.08	−0.02

*p < 0.05, **p < 0.001.

### Regression Analysis Using Health Self-Perception as a Dependent Variable and Sleep and Anxiety Scores as Independent Variables

Associations of HSP with sleep and anxiety scores, based on logistic regression analysis, are presented (see Supplementary Table S2). Among all participants, the HSP showed a significant positive relationship with the sleep score (β = 0.04; OR: 1.041; 95%CI: 1.001–1.083; p = 0.04), while it was negatively related to the STAI state score (β = −0.024; OR: 0.976; 95%CI: 0.953–0.999; p = 0.04). Furthermore, among female participants, HSP showed a significant negative association with sleep score (β = −0.07; OR: 0.93; 95%CI: 0.88–0.99; p = 0.02) and a significant positive association with STAI trait score (β = 0.04; OR: 1.04; 95%CI: 1.00–1.07; p = 0.03).

## Discussion

The objective of the present study was to determine the HSP and its association with anthropometric measurements, sleep quality, and anxiety levels among university students. About three-fifths of the participants reported positive HSP, which was dependent on sex and BMI. Female participants with positive HSP had higher sleep score and lower STAI state and trait scores. The HSP showed a positive relationship with sleep score and a negative relationship with STAI trait score.

The HSP was dependent on sex most of the students who perceived their health as negative were females (58.8%); this finding is supported by a study indicated that 33% of adolescent girls had negative HSP which was twice as high as boys [7]. For older adults, the average SRH score in a study done in China showed a substantial gender difference, with females having a lower SRH than males [35]. Similarly, another study reported that the association between sex and fair/poor self-rated health was statistically significant, with women reporting greater prevalence ratios of fair/poor self-rated health than men [36]. This difference in HSP between sexes was explained in a cohort study addressed the Eastern European countries, which is mostly existing due to cultural, social and economic factors, women face a variety of challenges, including solidarity, calendar management, diversity and equality work, and the welfare of friends and family (third shift), in addition to employment (first shift) and housework (second shift). While one of these jobs has the potential to cause gender difficulties, they all work together to create tension and discomfort. The responsibilities and lives of women might differ among cultures and generations. Hence, while designing interventions, it is important to consider the cultural context during design [36].

However, HSP was not dependent on age, marital status, educational level, and university major. This finding is somewhat surprising, as various prior studies have highlighted age and education level as impactful factors in HSP. For example, research conducted by Yoon et al. [25], indicated that older individuals typically report worse (SRH) in comparison to younger groups, likely due to their greater exposure to chronic illnesses and physical limitations that tend to accumulate with age. Additionally, Aguilar-Palacio et al. [4], found a positive correlation between educational achievement and enhanced health perception, noting that individuals with higher education levels are more likely to adopt health-promoting behaviors and seek medical care. Nonetheless, the lack of significance in the current study may stem from the uniformity of the sample, which primarily consists of young adults within a limited age range (18–25 years) who are comparatively healthy and less likely to be affected by chronic health issues. This age group may not yet face the health declines associated with aging, which could obscure any variations in health perception related to education or life stage. Furthermore, the lack of a significant association between HSP and enrollment in a health-related major is especially striking. This finding implies that academic exposure to health information does not always lead to an improved health perception, contrary to the results of Kim & Lee [34, 37]. Who found that students in health-related fields reported greater health awareness and superior self-perceived health due to their enhanced understanding of health risks and preventive measures. However, the findings of this study may indicate a disconnect between theoretical health knowledge and actual health behaviors among university students. This observation is consistent with results from Alzahrani et al, [38]. Who reported that medical and health science students in Saudi Arabia demonstrated an understanding of health risks but did not consistently engage in healthy lifestyle practices, showcasing a gap between knowledge and behavior. Additionally, cultural influences may also contribute to this discrepancy. In Middle Eastern cultures, where familial impact is significant, health perceptions might be more influenced by family expectations and community beliefs rather than academic education (Al-Yateem et al.,) [16]. This cultural backdrop could diminish the potential positive influence of health-related education on self-perception. Moreover, Lee et al. [37], found that health literacy is shaped not just by educational exposure but also by social interactions and community health awareness, suggesting that initiatives focusing on community health education could have a greater effect on changing health perceptions than purely classroom learning. These results point to the necessity for a more practical implementation of health education within health-related fields, stressing behavior change and lifestyle adjustments rather than exclusively theoretical knowledge. Incorporating practical health-promoting programs into university coursework, coupled with community engagement efforts, might significantly enhance the real-world influence of health education on students’ health perceptions.

A critical factor that contributes to a healthy lifestyle is sleep health, which is described as an integral part that reflects general health. The association of sleep quality with psychological symptoms among university students has been reported by previous researchers [39]. We found that those who positively perceived their health had higher scores for state anxiety as measured by STAI were notably higher in individuals with negative health self-perception (p = 0.002), especially among females (p = 0.001). This supports the findings of Hossain et al. [22]. Who indicated a strong correlation between high anxiety levels and poor health perception among university students. Which is in concordance with the previous study, reported that those with positive HSP had significantly higher sleep scores and lower STAI state scores indicating good quality of sleep and lower anxiety [37, 39], which may consequently affect the quality of life [37]. There it has been reported that better sleep quality, longer mean sleep duration and less fluctuation in sleep duration were all associated with greater life satisfaction in university students [40]. In addition, a previous study finding indicated that those who get better sleep also report feeling happier, more content with life, more wellbeing, and less stressed at work [41]. The differences observed between sexes imply that anxiety might have a more significant impact on how female students perceive their health. This finding is consistent with the Transactional Model of Stress and Coping [42], which suggests that an individual’s assessment of stress and their coping strategies can directly influence their evaluations of both mental and physical health.

Furthermore, duration and quality of sleep both had an impact on quality of life and life satisfaction [43]. Similar to our findings, Delmases et al. [9], assessed the relationship between sleep and HSP, demonstrating that poor sleep was strongly associated with poor self-perceived health status. In a recent systematic review, the relationship between sleep quality, sleep duration and insomnia with SRH was examined, and found that the sleep durations of 8 h or more and less than 8 h are linked to poor SRH. However, compared to prolonged sleep duration, shorter sleep duration was linked to a higher likelihood of having poor SRH [44]. Similarly, a previous cross-sectional study, showed that short (<6 h) and long sleep duration >10 h was associated with poor SRH [45]. The findings of previous meta-analysis and systemic reviews have also shown that sleep length have significant effects on different dimensions of health and demonstrated that both short and long sleep duration was associated with increased risk of mortality, stroke, coronary heart diseases and increased risk of obesity and overweight [46, 47].

Thus, it can be suggested that sleep health and habits should be included as a crucial modifiable health risk factor that can have a high impact on a healthy lifestyle. This aligns with the findings of a study demonstrated that poor or inadequate sleep was strongly associated with a high frequency of mental distress in adults [48]. In contrast to our findings, a previous showed that poor sleep (29.1%) was found to be significantly higher in women with poor self-rated health status. It is noteworthy that poor sleep quality is a global health concern and is associated with HSP which contributes to diseases and poor health outcome [49], and may affect the academic performance of the students [37]. Therefore, it is recommended that designed interventions should include educational sessions to raise awareness to improve sleep quality among university students. Self-perception of physical health is associated with anxiety and depression among university students and reported that anxiety scores having a significant negative correlation with HSP. This is similar to a recent study found that poor SRH is significantly associated with anxiety among university students and those who had negative HSP had higher chances to develop depression and anxiety [22, 50]. Boldt et al. [50], also suggest that negative HSP was strongly associated with diagnosed or present anxiety/depression. In addition, SRH was found to be also a predictor of the onset of anxiety and depression conditions [51].

Regarding psychological parameters it is worth noting that our results showed that females had a higher median STAI state score compared to males (*p* = 0.036), suggesting heightened situational anxiety among female students. This finding is consistent with prior research indicating that women are more prone to anxiety due to sociocultural pressures and biological susceptibility [52]. Conversely, males reported a significantly higher STAI trait score (*p* = 0.005), This indicates that females in general, are more inclined than males to worry (ruminate), and they also appear to perceive dangers as more unpredictable and unmanageable. In addition, women appear to be more sensitive to worry than men [52]. Such high STAI state anxiety puts individuals at a strong risk for poor health assessment in health profession students which can lead to poor health-related quality of life [53]. Regarding the higher STAI trait score in males, it could be attributed to collectivist culture in Arab and western society, in which men from early age are frequently socialized to exhibit behaviors that are viewed as (and recognized as) traditionally masculine. These behaviors include emotional restrictiveness and heterosexual presentation which were all associated with higher levels of anxiety in youngsters and adults [54]. In contrast to our finding, Martinez et al [55], found that there is no significant differences in state anxiety and trait anxiety (p > 0.05) were observed according to sex, while other studies supported our findings revealing that females had higher trait anxiety scores [56, 57].

Interestingly, sleep scores did not differ significantly between males and females (*p* = 0.16), contradicting some previous studies which suggested that females generally report poorer sleep quality [39]. This could be attributed to cultural or environmental factors unique to the UAE, suggesting that future research might benefit from exploring region-specific sleep patterns in greater detail.

Moreover, in regards to body composition, sleep quality and anxiety, although waist circumference, fat mass, and visceral fat rating did not exhibit any significant relationships with sleep or anxiety scores in negative HSP group, there was a slight, non-significant, positive correlation between waist circumference and sleep score (r = 0.13). This might suggest that central adiposity is not the main factor affecting sleep quality in this subgroup, differing from results in clinical populations where obesity is strongly associated with poor sleep [58]. The noted disconnect could indicate a psychological bias, where perceptions of negative health are influenced more by subjective feelings of wellbeing than by objective physical measures Furthermore, fat mass (r = −0.13, p < 0.05) and visceral fat rating (r = −0.15, p < 0.05) showed a negative correlation with sleep scores in positive HSP group, suggesting that a healthier body composition is linked to enhanced sleep quality among individuals who view their health positively. This supports previous research indicating that a leaner body composition and decreased fat accumulation lead to better sleep quality and fewer sleep disruptions [59]. In addition, waist circumference demonstrated a positive correlation with STAI State scores and an inverse relationship with STAI Trait scores within the group having a positive health self-perception (p < 0.05), implying that central fat accumulation may worsen situational anxiety in students who perceive their health positively. This corresponds with the research findings of Griffiths et al. [60]. Which indicated that even those students with a generally positive perception of their health may still experience anxiety associated with weight gain and central fat distribution.

Our study showed that male students showed significantly higher values in waist circumference (WC), fat-free mass (FFM), muscle mass, visceral fat rating (VFR), actual BMI, and perceived BMI compared to their female counterparts (*p* < 0.001). Conversely, female students exhibited higher levels of percent body fat (PBF) and fat mass (*p* < 0.001), which aligns with previous studies indicating that females tend to have higher body fat percentages due to physiological and hormonal differences [7]. This body composition difference is important as it may influence health self-perception (HSP), as we reported that university students showed better HSP when they had lower PBF, FM and VFR, and higher FFM, these observations were consistent with previous literature reporting that positive HSP was more prevalent among obese adolescents following weight management approaches to improve anthropometric parameters and body composition [61].

The differences noted between males and females in various physical and psychological metrics may indicate not just biological variations but also cultural impacts on health perceptions and mental wellbeing. This is backed by the Biopsychosocial Model, which suggests that health results arise from a combination of biological, psychological, and social elements. These results highlight the necessity of tailored health interventions that address gender-specific vulnerabilities and societal expectations, which may affect HSP differently in male and female students [62].

Interestingly, we identified that PBF, FM and WC were significantly higher in the negative HSP than positive HSP group (p < 0.001), especially among males, indicating the higher incidence of overweight and obesity changes body image satisfaction. This observation aligns with prior study indicating that higher levels of body fat and central obesity are closely associated with poorer health self-perception [63]. A larger WC, which indicates central obesity, is widely recognized as a risk factor for metabolic issues and is often connected to negative health perceptions due to its visible effect and societal stigma related to abdominal obesity [64]. PBF, an important measure of body composition, was significantly elevated in those with negative HSP, suggesting a higher fat accumulation in relation to lean body mass. This correlation may arise from dissatisfaction with body image, since higher PBF is frequently associated with negative self-image, particularly among young adults sensitive to societal norms regarding appearance. [65]. Male participants but not females, with negative HSP had significantly higher actual and perceived BMI compared to those with positive HSP (p < 0.001), indicating a potential link between self-perception and body image issues. This result is consistent with recent research suggesting that body image challenges are not solely a concern for females but are increasingly prevalent among males, influenced by societal pressures and ideals surrounding muscularity and leanness [66, 67]. Unlike females, who may concentrate more on weight and fat distribution, males often associate larger waist circumference and elevated BMI with poor health and diminished physical fitness, impacting their self-assessment of health negatively [68]. This implies that health self-perception among males could be more intimately tied to observable physical characteristics, such as abdominal fat and total body mass, which can be easily acknowledged and socially assessed. Conversely, females exhibited less variation in body composition metrics between those with positive and negative HSP, suggesting that psychological elements like anxiety and sleep issues may have a more substantial influence on their health perceptions than purely physical measures. This belief is supported by findings that indicate women are usually more affected by psychological stressors, such as anxiety and sleep quality, when judging their health condition [69, 70].

Additionally, students with negative HSP reported higher visceral fat ratings (VFR), which is particularly alarming because increased VFR has been independently associated with a higher risk of metabolic syndrome, cardiovascular diseases, and insulin resistance [27]. Visceral fat is understood to be more metabolically active than subcutaneous fat, contributing to chronic inflammation and a greater risk of cardiometabolic complications [71]. This correlation between negative HSP and elevated VFR implies that students with unfavorable health perceptions might face a higher risk of long-term health complications, underscoring the urgent need for early interventions.

Moreover, Previous studies also reveal that body image and physical health are positively associated with weight, BMI and FM as well as other body composition indicators that directly influence health status [63, 72]. Intriguingly, we observed that the positive HSP group had better body composition indicators than the negative HSP group which supports the concept that appropriate body indicators may contribute to healthy physical status.

These results highlight the vital importance of considering body composition, rather than just weight, when assessing health self-perception. Tailored health interventions that address both physical and psychological aspects are crucial for university students. Potential strategies may include body composition assessments, nutrition education, and mental health resources, particularly aimed at alleviating anxiety and enhancing body image. Addressing these factors collectively could improve students’ health perceptions and potentially decrease their risk of metabolic and psychological health challenges.

### Limitations of the Study

The study has some limitations, such as cross-sectional design which fails to establish causal relationships. Moreover, data were self-reported for sleep quality and anxiety pattern, this may add to bias, as people report their own experiences and may be difficult for individuals to accurately recall. Additionally, the sample size was relatively small, hence future large-scale studies regarding HSP, particularly in females may be warranted. Lastly, the age distribution of the sample, which was largely composed of people under 20, is another limitation. This age range was specifically chosen because early stages of adulthood are crucial for the formation of one’s self-perception and body image. However, their answers might not accurately represent the views of older populations with more stable self-concepts because these people are still going through major psychological and physical changes. Future research could get a more comprehensive understanding and enable significant cross-developmental comparisons by involving postgraduate or older students. To overcome these limitations, subsequent research should embrace longitudinal methodologies to enhance the understanding of the causal relationships and long-term effects of sleep quality and anxiety on individuals’ perceptions of their health. Incorporating objective assessments, such as actigraphy for monitoring sleep and clinical evaluations for anxiety, would yield a more precise comprehension of these factors. Furthermore, broadening the research to encompass multiple universities and a more diverse age demographic would improve the applicability of the results. Implementing cross-cultural research would aid in discovering whether the identified relationships are specific to the UAE or if they hold true across various cultural environments. In addition, creating health intervention initiatives within university contexts that aim to enhance sleep quality, address anxiety, and encourage healthy body composition could positively impact students’ health perceptions. Ultimately, the results underline the necessity for policies that tackle mental health and sleep hygiene within educational institutions, fostering a comprehensive approach to student wellness.

### Conclusion and Significance

The current study provides significant insights into the relationship between health self-perception (HSP), sleep quality, anxiety levels, and body composition among university students in the United Arab Emirates. One of the primary strengths of the study is its focus on a culturally specific context that is often underrepresented in global health research. By examining a diverse student population within the UAE, the study contributes to a deeper understanding of how cultural and environmental factors influence HSP. Furthermore, the use of a validated questionnaire for sleep quality and anxiety, alongside objective measurements of body composition, strengthens the reliability of the findings. This comprehensive approach allows for a robust exploration of both subjective perceptions of health and objective health indicators, bridging the gap between self-reported health data and measurable health outcomes. Additionally, the large sample size of 390 participants provides a substantial dataset for statistical analysis, enhancing the study’s generalizability within the student population in the region. The cross-sectional design also enables the identification of significant associations between variables, highlighting the critical role that sleep quality and anxiety play in students’ perceptions of their health. These findings suggest important implications for university health programs and policies aimed at improving student wellbeing. The findings of this study highlighted that sex is a good predictor for HSP. A high prevalence of negative HSP was observed particularly in female university students. STAI state score was significantly higher whereas the sleep score was significantly lower in the negative HSP group compared to the positive HSP. This indicates that good quality of sleep and low anxiety levels represent positive perceptions of health. Moreover, abnormal body composition indicators adversely affect the HSP. Therefore, academic settings should encourage students to follow a healthy diet, regular exercise, and maintain good sleeping patterns to reduce their anxiety. Additionally, health professionals should pay more attention to young adults who are overweight and obese with high-body fat percentage, lower FFM, and high anxiety as they are at risk for negative HSP. More screening and awareness programs should be held for students at Universities across the UAE.
