# Supplementary material for: Sex Based Comparison of Health Self-Perception, Sleep, Anxiety, and Body Composition Among University Students
Source: Int J Public Health. 2025 Sep 26;70:1608551. doi: 10.3389/ijph.2025.1608551 (PMC12510905; doi:10.3389/ijph.2025.1608551)
Supplement: Supplementary file 1 [file Supplementaryfile1.docx]

| **Supplementary Table 1.** Association of health self-perception with socio-demographic characteristics | | | | | | | | | | |
| --- | --- | --- | --- | --- | --- | --- | --- | --- | --- | --- |
| **Variables** | |  | **Health Self-Perception** | | |  |  | **95% CI** | |  |
|  |  |  | **Positive** (n=230)  **%(N)** |  | **Negative** (n=160)  **%(N)** |  | **OR** | **Lower** | **Upper** | **p-value** |
| **Age (years)** | |  |  |  |  |  |  |  |  |  |
|  | <20 |  | 65.7(151) |  | 66.9(107) |  | 0.94 | 0.61 | 1.45 | 0.802 |
|  | 21-25 |  | 34.3(79) |  | 33.1(53) |  |  |  |  |  |
| **Sex** | |  |  |  |  |  |  |  |  |  |
|  | Female |  | 42.6(98) |  | 58.8(94) |  | 0.52 | 0.34 | 0.78 | 0.002 |
|  | Male |  | 57.4(132) |  | 41.2(66) |  |  |  |  |  |
| **BMI** |  |  |  |  |  |  |  |  |  |  |
|  | Abnormal |  | 40.0(92) |  | 55.0(88) |  | 0.54 | 0.36 | 0.82 | 0.003 |
|  | Normal |  | 60.0(138) |  | 45.0(72) |  |  |  |  |  |
| **Marital status** | |  |  |  |  |  |  |  |  |  |
|  | Single |  | 97.8(225) |  | 96.9(155) |  | 1.45 | 0.41 | 5.00 | 0.559 |
|  | Married |  | 2.2(5) |  | 3.1(5) |  |  |  |  |  |
| **Are you in a health-related major?** | | | |  |  |  |  |  |  |  |
|  | No |  | 43.5(100) |  | 47.5(76) |  | 0.85 | 0.58 | 1.27 | 0.432 |
|  | Yes |  | 56.5(130) |  | 52.5(84) |  |  |  |  |  |
| **Educational level** | | | |  |  |  |  |  |  |  |
|  | First Year |  | 37(85) |  | 34.5(55) |  | - | - | - | 0.755 |
|  | Second Year |  | 23.5(54) |  | 26.9(43) |  |  |  |  |  |
|  | Third Year |  | 12.6(29) |  | 15.6(25) |  |  |  |  |  |
|  | Fourth Year |  | 22.6(52) |  | 20(32) |  |  |  |  |  |
|  | Fourth Year+ |  | 4.3(10) |  | 3.1(5) |  |  |  |  |  |

Sharjah, United Arab Emirates, 2020

| **Supplementary Table 2.** Regression analysis using health self-perception as a dependent variable and sleep and anxiety scores as independent variables | | | | | | | | | | | | | | | |
| --- | --- | --- | --- | --- | --- | --- | --- | --- | --- | --- | --- | --- | --- | --- | --- |
|  | **All** | | | | | **Males** | | | | | **Females** | | | | |
|  | **β** | **P-value** | **OR** | **95% CI** | | **β** | **P-value** | **OR** | **95% CI** | | **β** | **P-value** | **OR** | **95% CI** | |
|  |  |  |  | **Lower** | **Upper** |  |  |  | **Lower** | **Upper** |  |  |  | **Lower** | **Upper** |
| Sleep score | 0.040 | 0.04 | 1.04 | 1.00 | 1.08 | -0.01 | 0.70 | 0.89 | 0.93 | 1.04 | -0.07 | 0.02 | 0.93 | 0.88 | 0.99 |
| STAI State Score | -0.024 | 0.04 | 0.97 | 0.95 | 0.99 | 0.02 | 0.22 | 1.02 | 0.98 | 1.06 | 0.2 | 0.24 | 1.02 | 0.98 | 1.05 |
| STAI Trait Score | -0.009 | 0.43 | 0.99 | 0.96 | 1.01 | -0.004 | 0.77 | 0.99 | 0.96 | 1.02 | 0.04 | 0.03 | 1.04 | 1.00 | 1.07 |
| Positive health self-perception coded 2  Negative health self-perception coded 1 | | | | | | | | | | | | | | | |

Sharjah, United Arab Emirates, 2020
